# Anti-KIT designer T cells for the treatment of gastrointestinal stromal tumor

**DOI:** 10.1186/1479-5876-11-46

**Published:** 2013-02-21

**Authors:** Steven C Katz, Rachel A Burga, Seema Naheed, Lauren A Licata, Mitchell Thorn, Doreen Osgood, Cang T Nguyen, N Joseph Espat, Jonathan A Fletcher, Richard P Junghans

**Affiliations:** 1 Roger Williams Medical Center, Department of Surgery, Providence, RI/Boston University School of Medicine, Boston, MA, USA; 2 Roger Williams Medical Center, Department of Medicine, Providence, RI/Boston University School of Medicine, Boston, MA, USA; 3 Brigham and Women's Hospital and Harvard Medical School, Boston, MA, USA

**Keywords:** GIST, Adoptive cell therapy, Immunotherapy, and Designer T cells

## Abstract

**Background:**

Imatinib mesylate is an effective treatment for metastatic gastrointestinal stromal tumor (GIST). However, most patients eventually develop resistance and there are few other treatment options. Immunotherapy using genetically modified or designer T cells (dTc) has gained increased attention for several malignancies in recent years. The aims of this study were to develop and test novel anti-KIT dTc engineered to target GIST cells.

**Methods:**

Human anti-KIT dTc were created by retroviral transduction with novel chimeric immune receptors (CIR). The gene for stem cell factor (SCF), the natural ligand for KIT, was cloned into 1^st^ generation (SCF-CD3ζ, 1st gen) and 2^nd^ generation (SCF-CD28-CD3ζ, 2nd gen) CIR constructs*. In vitro* dTc proliferation and tumoricidal capacity in the presence of KIT+ tumor cells were measured. *In vivo* assessment of dTc anti-tumor efficacy was performed by treating immunodeficient mice harboring subcutaneous GIST xenografts with dTc tail vein infusions.

**Results:**

We successfully produced the 1^st^ and 2^nd^ gen anti-KIT CIR and transduced murine and human T cells. Average transduction efficiencies for human 1^st^ and 2^nd^ gen dTc were 50% and 42%. When co-cultured with KIT+ tumor cells, both 1^st^ and 2^nd^ gen dTc proliferated and produced IFNγ. Human anti-KIT dTc were efficient at lysing GIST *in vitro* compared to untransduced T cells. In mice with established GIST xenografts, treatment with either 1^st^ or 2^nd^ gen human anti-KIT dTc led to significant reductions in tumor growth rates.

**Conclusions:**

We have constructed a novel anti-KIT CIR for production of dTc that possess specific activity against KIT+ GIST *in vitro* and *in vivo*. Further studies are warranted to evaluate the therapeutic potential and safety of anti-KIT dTc.

## Background

Gastrointestinal stromal tumor (GIST) is the most common GI mesenchymal neoplasm and nearly all GIST express KIT (CD117) [1]. Imatinib mesylate has been demonstrated to significantly prolong disease-free survival in the adjuvant setting and for patients with disseminated GIST [2,3]. Unfortunately, the majority of patients with metastatic GIST who are treated with imatinib develop resistance and subsequently progressive disease [4]. Therapeutic options are limited for patients who develop advanced GIST unresponsive to tyrosine kinase inhibitor (TKI) therapy. In an effort to address this clinical need, we sought to develop a T cell-based therapy for the treatment of KIT+ tumors including GIST.

Cell-based immunotherapy using tumor infiltrating lymphocytes (TIL) has shown success for specific diseases [5]. The potential impact of TIL therapy is limited by the inability to isolate TIL from the majority of patients with solid tumors [6]. The genetically modified or designer T cell (dTc) strategy allows for the production of tumor-specific lymphocytes for any patient with a suitable target tumor antigen. Lymphocytes are isolated from peripheral blood and activated prior to retroviral transduction with a chimeric immune receptor (CIR) gene [7]. Expression of CIR on the surface of modified T cells allows for highly specific recognition of tumor cells expressing the cognate antigenic moiety. Retrovirus mediated introduction of tumor specific CIR into human T cells has resulted in dTc capable of activation, cytokine secretion, and target cell lysis [8-10]. Clinical success has recently been reported using dTc for the treatment of soft tissue sarcoma, melanoma, and leukemia [11,12]. We are not aware of prior efforts to treat GIST using dTc.

CIR typically exploit immunoglobulin or T cell receptor based specificity to target tumor antigens. Using an alternative strategy, we engineered a CIR that contains the natural ligand for KIT, which allows for recognition of KIT+ tumor cells. KIT-ligand (KL) or stem cell factor (SCF) was fused to the CD3ζ chain component of the T cell receptor (1st generation, 1^st^ gen) or CD3ζ + the CD28 co-stimulatory molecule (2nd generation, 2^nd^ gen). The 2^nd^ gen dTc express the construct that targets KIT+ tumors while, at the same time, integrating CD28 co-stimulatory signals. 1^st^ and 2^nd^ gen dTc were produced and tested *in vitro* and *in vivo* to demonstrate their efficacy in destroying KIT+ tumor cells. The present report demonstrates encouraging initial results for anti-KIT dTc and provides the rationale for further pre-clinical testing of this novel immunotherapeutic anti-tumor agent.

## Methods

### Retroviral vector construction

First and second generation anti-KIT CIR were re-engineered from the anti-CEA retroviral vector expression constructs previously described [7]. The extracellular domain of cKIT ligand ([Genbank:BC069733.1], cDNA clone MGC:97379) spanning the N-terminal start codon to the transmembrane start was PCR amplified from ATCC clone 010560371 using primers incorporating NcoI and BamHI restriction sites and cloned in-frame to replace the anti-CEA extracellular domain.

Extracellular domain of cKIT ligand:

(5’-gattccaggaattgatttccccatggcaaagaagacacaaacttg-3’

5’-ctaagctctagccaattgaattggatccgtgtaggctggagtctcc-3’)

### Designer T cell production

Human peripheral blood mononuclear cells (PBMC) were obtained from random donor whole blood filtrate (Rhode Island Blood Center, Providence, RI). Blood filters were washed with sterile PBS (Cellgro, Manassas, VA) and PBMC were isolated by density gradient separation with Histopaque (Sigma-Aldrich, St. Louis, MO) according to manufacturer directions. PBMC were seeded at a density of 2 × 10^6^ cells/ml, and activated on anti-CD3 coated (OKT3, eBioscience, San Diego, CA) 750 ml flasks with 2 ug/mL anti-CD28 (CD28.2, eBioscience) and 300 U/mL of human IL-2 in AIM V medium (Invitrogen, Grand Island, NY) supplemented with 5% heat inactivated sterile human serum (Valley Biomedical, Winchester, VA). 293T-HEK phoenix amphotropic cells (Orbigen, Allele Biotechnology, San Diego, CA) were transfected with 50 μg 1^st^ or 2^nd^ gen c-KIT ligand CIR retroviral plasmid using LipoD283 (SignaGen Laboratories, Rockville, MD). Viral supernatant was harvested for transduction of NIH-3T3 PG13 retrovirus packaging cells (ATCC: CRL-10686) cells that had reached 80% confluence. PG13 cells were cultured at 37°C and supernatant was harvested and filtered through 0.45 μm filters (Corning, Corning NY) when cells reached 80% confluence. After 24-48 hours of culture, PBMC were seeded on retronectin-coated (20 ug/mL, Takara Bio, Otsu, Shiga, Japan) wells of a 6-well plate and were transduced with viral supernatant as described to create designer T cells [7]. Cells were transduced with supernatant containing either anti-KIT CIR vector (1^st^ gen) or anti-KIT CIR vector with additional CD28 moiety (2^nd^ gen). Transduced T cells were maintained in AIM V medium supplemented with 5% heat inactivated sterile human serum and 100 IU/ml IL-2. Expression of KIT-specific CIR on designer T cells was evaluated by flow cytometric analysis of staining with anti-SCF mAb (Reprokine, Valley Cottage, NY) conjugated to APC (Chromaprobe, Maryland Hts, MO). Cells were also stained with antibodies against human CD3 (Sk7), CD4 (RPA-T4), CD8 (SK1), CD62L, CD45RO, CD197 (CCR7, 150503), and CD25 (M-A251), which were conjugated to FITC, PE, PerCP, APC, APC-Cy7, or Pe-Cy7 (BD Biosciences, Franklin Lakes NJ). For FoxP3 intracellular staining, samples were fixed, permeabilized, and stained with FoxP3 conjugated to PE as per manufacturer’s protocol (BD).

### Cell proliferation assay

Flow cytometry-based division assays were performed to analyze the proliferation of 1st and 2nd gen dTc in response to stimulation by KIT+ human GIST cell lines GIST882 [13] and GIST48, [14] both of which contain oncogenic KIT mutations. GIST882 was established from an untreated GIST, whereas GIST48 was established from a kinase-inhibitor resistant GIST which was progressing clinically after initial response to imatinib therapy. DTc were labeled with 1 μM carboxyfluorescein diacetate succinimidyl ester (CFSE, Invitrogen) and were added at a 4:1 ratio with KIT+ GIST 882 and GIST 48 cells in a 96-well round-bottom plate, with 1 × 10^5^ dTc added per well. GIST48B cells [15], which have minimal KIT surface expression, were used as a negative control initially. MC38 murine colorectal carcinoma cells were also used as negative controls to ensure complete absence of human KIT on the cell surface. Tumor cells were irradiated at 5000 rad. Co-culture was incubated for 5 days, at which point supernatant was isolated and cells were analyzed by flow cytometry. Supernatant was analyzed by cytometric bead array for IFN-γ levels (BD Biosciences). Cytokine production results were also quantified by human IFN-γ ELISA assay for confirmation (Biolegend).

### Cytotoxicity assays

1^st^ and 2^nd^ gen dTc were cultured with KIT+ GIST 882 or GIST 48 cells in order to evaluate their cytotoxic ability in an LDH assay (Roche, Indianapolic, IN) performed according to the manufacturer’s protocol. Tumor cells were irradiated at 5000 G for 50 minutes. Cytotoxic ability was evaluated for 1^st^ gen dTc, 2^nd^ gen dTc, and untransduced human T cells, which were added at various effector-to-target ratios. Cytotoxicity results from the LDH assay were further confirmed by flow cytometric analysis of tumor cell death. GIST cells were irradiated as previously described and labeled with CFSE while dTc were unstained; loss of CFSE+ cells was analyzed with flow cytometry.

### *In vivo* tumor studies

Six-week-old male immunodeficient mice (NU/J) were purchased from Jackson Laboratories (Bar Harbor, ME) and experiments were conducted in compliance with the guidelines of the Roger Williams Medical Center Institutional Animal Care and Use Committee. The GIST cell lines were maintained at 37°C in IMDM with 1% l-glutamine (Invitrogen) supplemented with 15% FBS, and 1% Penicillin/Streptomycin/Amphomycin (Cellgro). We administered bilateral subcutaneous flank injections of 3 × 10^7^ KIT+ GIST 882 cells in 200 ul sterile PBS. DTc or untransduced human T cells were injected (1 × 10^7^ in 200 ul PBS) via tail vein. For experimental groups with IL-2, Alzet 7-day micro-osmotic pumps (Durcet, Cupertino CA) were filled with IL-2 according to the manufacturer’s protocol and implanted subcutaneously. Pumps were set to deliver at a rate of 10,000 IU/h (550 pg/h). Tumors were measured in two dimensions with calipers, and measurements were obtained daily from the time of T cell injection until the conclusion of the study. The average of right and left flank tumors was used for each mouse, and measurements were normalized to initial tumor size. After sacrifice, tumors were excised and sent to the University of Massachusetts, Worchester Medical Center Experimental Pathology Service Core, for histological sectioning and staining. Sections were stained for routine H&E and anti-CD3 immunohistochemistry. Slides were analyzed at the Pathology Department at Roger Williams Medical Center, and photographs were taken under 10x and 40x magnification.

### Statistics

Statistics were calculated using GraphPad Prism V5.00 for Windows (GraphPad Software, San Diego, CA). Statistical significance for proliferation and cytotoxicity assays was determined using the two-tailed Student *t* test, and values with p≤0.05 were classified statistically significant. Tumor size median values are presented and logistic regression was used to compare growth curve slope and elevation among groups. Cell proliferation analysis with calculation of division peaks was performed using FlowJo software (Treestar, Ashland, OR).

## Results

### Engineering of anti-KIT chimeric immune receptors and production of designer T cells

**Figure 1:**
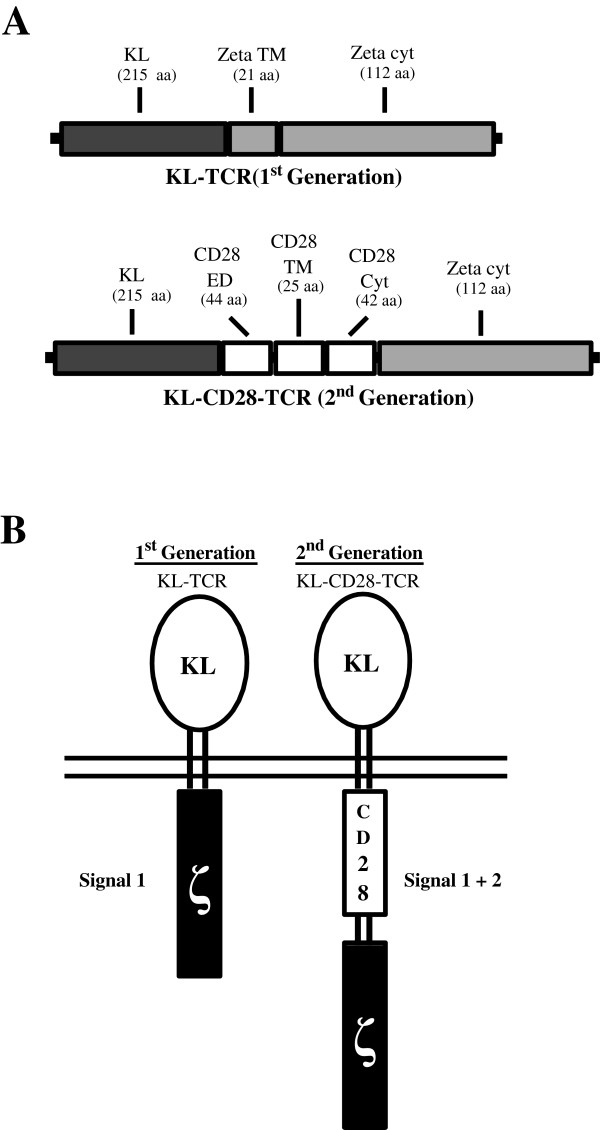
**Structure of anti-KIT chimeric immune receptor.** (**A**) Schematic diagram of 1^st^ and 2^nd^ generation CIR genetic constructs. (**B**) Structure of 1^st^ and 2^nd^ generation CIR. Anti-KIT CIRs were re-engineered from anti-CEA retroviral vector constructs, whereby the extracellular domain of kit ligand (KL) was amplified and cloned to replace the anti-CEA extracellular domain.

Our aim for this study was to construct and test the function of KIT-specific CIR expressed by human peripheral blood T cells for pre-clinical development. We based our anti-KIT CIR construct on our pre-existing anti-CEA format [7]. The anti-CEA sFv fragment was replaced with the extracellular domain of KL. 1^st^ and 2^nd^ gen constructs were prepared (Figure 1A). The 2^nd^ gen construct contains CD28 to provide co-stimulation. The KL component is expressed on the extracellular aspect of the CIR to enable interaction with KIT on the surface of target tumor cells (Figure 1B). We confirmed our constructs by direct DNA sequencing prior to transduction of activated lymphocytes (data not shown).

**Figure 2:**
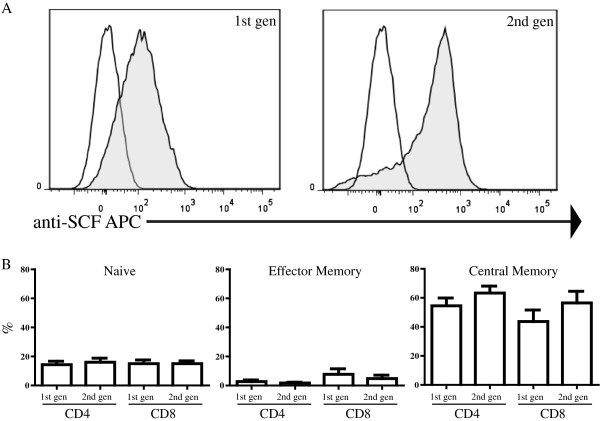
**Transduction efficiency and phenotype of human anti-KIT designer T cells (dTc).** (**A**) PBMC were isolated and primary human T cells activated and transduced with retrovirus expressing KIT-specific CIRs. Shaded histograms represent dTc and open histograms represent untransduced cells. (**B**) Flow cytometric analysis of the phenotype of 1^st^ and 2^nd^ generation dTc demonstrated that T cells (CD4+ and CD8+ T cells) of a central memory phenotype (CD45RO+CD62L+CCR7+) were in the majority. Naïve T cells were defined as (CD45RO-CD62L+). Data are representative of three or more repetitions.

Following activation and transduction, CIR expression was confirmed by flow cytometry with an anti-KL antibody. Retroviral transduction of activated murine splenocytes (not shown), which we used as a preliminary assessment, resulted in mean 1^st^ and 2^nd^ gen CIR expression rates of 27% (range, 16–41). Following optimization of our protocol, transduction of activated human PBMC (Figure 2A) yielded mean transduction rates of 50% (range, 33-74) and 42% (range, 24–62) for 1^st^ and 2^nd^ gen human dTc respectively, with no significant difference between the two CIR versions (p=0.67). After stimulation of PBMC with anti-CD3, anti-CD28 and IL2, >70% of the cells were CD3+ (data not shown) and a central memory phenotype (CD45RO+CD62L+ CCR7+) predominated (Figure 2B) for both CD4+ and CD8+ T cells. Fewer than 30% of cells had a naïve (CD45RO-CD62L+) or effector memory (CD45RO+CD62L+CCR7-) phenotype, and less than 10% of transduced T cells from both generations had a regulatory T cell phenotype (CD25+FoxP3+) with no difference between the groups (data not shown). For 1^st^ gen dTc, 33.4% of T cells were CD4+CD8- and 52.7% were CD8+CD4-, while the corresponding values for 2^nd^ gen dTc were 35.5% and 52.6%. The CD4:CD8 ratio did not change after transduction or exposure to KIT+ tumor (not shown). For all subsequent experiments, dTc were used in bulk, without fractionating by CD4 or CD8 expression, in keeping with current clinical practice for dTc infusions.

### Proliferation of anti-KIT dTc in the presence of KIT+ tumor cells

**Figure 3:**
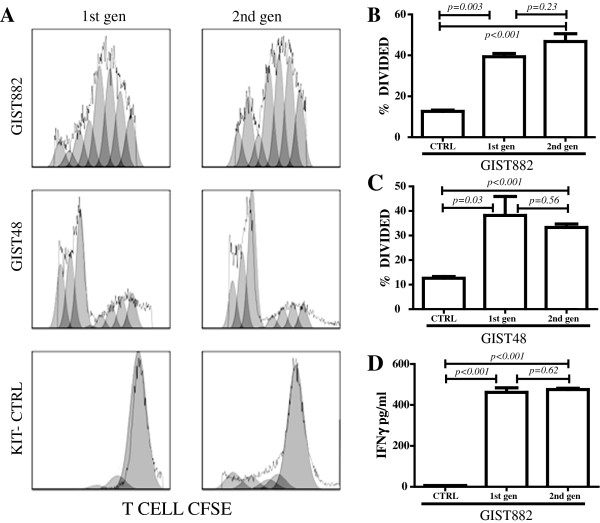
**Human anti-KIT dTc retain proliferative ability *in vitro*.** 1^st^ and 2^nd^ gen dTc were stimulated with human KIT+ GIST cell lines, GIST 882 and GIST 48. T cells were stained with CFSE and proliferation assessed by gating on CD3+ cells to measure CFSE dilution (**A-C**) and KIT- cells were used as a control. CIR- T cells did not proliferate in the presence of KIT+ tumor (not shown). (**D**) To confirm dTc activation by KIT+ GIST cell lines, IFNγ production was measured by ELISA, and was found to be in the range of 462-475 pg/ml, while that of untransduced CIR- T cells (CTRL) was negligible. Data are representative of three or more repetitions.

To test the proliferative capacity of human T cells expressing anti-KIT CIR, we cultured the dTc in the presence of two human KIT+ GIST cell lines, GIST882 and GIST48 [14,16]. In the presence of GIST882 and GIST48, dTc expressing either the 1^st^ or 2^nd^ gen anti-KIT CIR proliferated to a greater extent when compared to control groups (CTRL) as determined by CFSE dilution (Figure 3A). When cultured with GIST882, 39% of the 1^st^ gen and 47% of the 2^nd^ gen dTc divided (p<0.001 compared to CTRL), with no significant difference between the two CIR formats (p=0.23, Figure 3B). Likewise, in the presence of imatinib resistant GIST48 cells, 33–38% of the dTc divided after 3 days in culture which was significantly higher than CTRL cells (p≤0.03 compared to CTRL), with no significant difference between the two CIR formats (p=0.56, Figure 3C). The requirement of KIT+ tumor cells for dTc proliferation was confirmed by the minimal proliferation that resulted when culturing dTc in the presence of KIT- GIST 48B cells, and CIR- activated T cells did not proliferate in the presence of KIT+ tumor (data not shown). IFNγ production confirmed dTc activation by KIT+ tumor and was found to be in the range of 462-475 pg/ml, while production by CIR- T cells was negligible (p<0.001, Figure 3D). Co-culture of anti-KIT dTc with KIT- control tumor cells did not result in significant IFNγ production (data not shown).

### Lysis of KIT+ tumor cells by anti-KIT designer T cells

**Figure 4:**
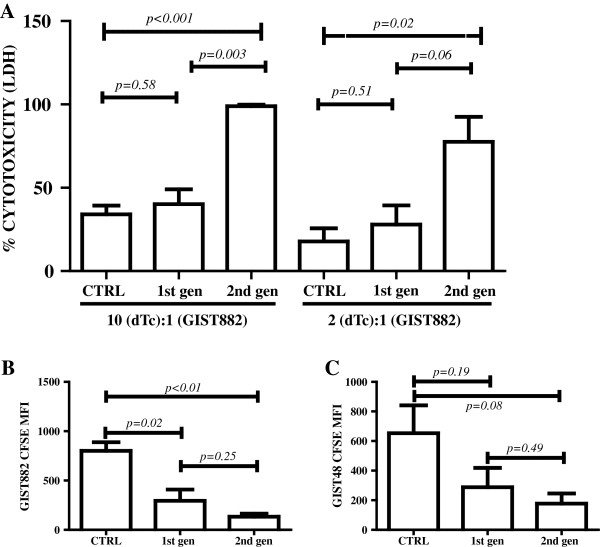
**Ability of human anti-KIT dTc to effectively lyse KIT+ tumor cells.** (**A**) LDH assay to evaluate enzymatic release following tumor cell lysis was performed on supernatant from co-culturing of 1^st^ and 2^nd^ generation dTc with irradiated GIST 882 cells. Maximal release was defined by the highest experimental values. (**B-C**) To confirm the cytotoxic ability of dTc, irradiated GIST 882 or GIST 48 cells were labeled with CFSE and cultured with unlabelled T cells. Tumor cell death was quantified by measuring the decrease in CFSE fluorescence by gating on remaining live cells. Data are representative of three or more repetitions.

The hallmark of effective adoptive cellular immunotherapy is the ability of the product to lyse tumor cells in a specific fashion. To this end, we performed *in vitro* assays to determine if dTc expressing anti-KIT CIR were able to destroy GIST cells. We demonstrated that 2^nd^ gen dTc effectively lysed KIT+ tumor and were more effective than the 1^st^ gen format by LDH release (Figure 4A). To confirm our findings, we mixed CFSE-labeled irradiated tumor cells with unlabeled T cells. Tumor cell loss or death was measured by quantifying the decrease in CFSE fluorescence from remaining live cells. When compared to CTRL cells, 1^st^ gen and 2^nd^ gen dTc mediated significant decreases in the level of CFSE fluorescence and hence number of live tumor cells (Figures 4B,C). Having demonstrated that the anti-KIT dTc were stimulated to divide *in vitro* in response to KIT+ tumor and lyse KIT+ targets, we sought to measure the *in vivo* efficacy.

### *In vivo* assessment of anti-KIT dTc

**Figure 5:**
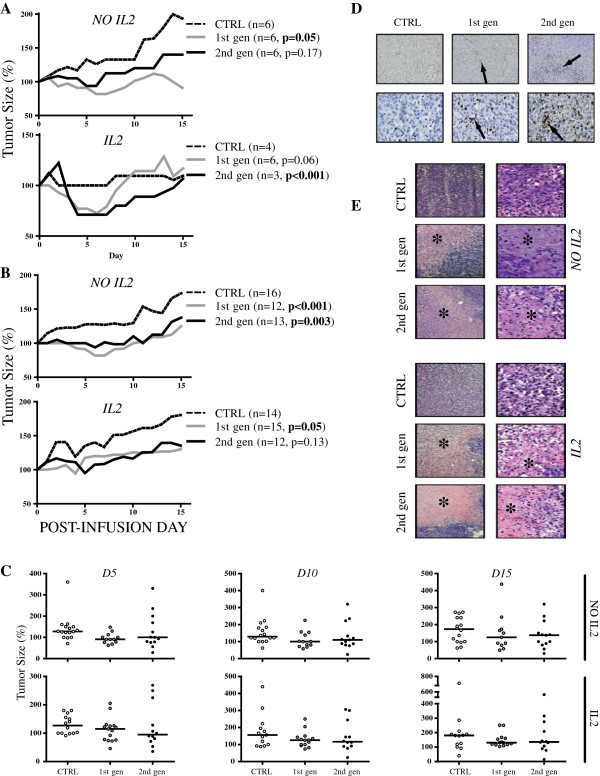
***In vivo *testing of human anti-KIT designer T cells.** We used a subcutaneous xenograft model whereby GIST 882 cells were injected subcutaneously into immunodeficient mice prior to treatment with human anti-KIT dTc. Median results from a single representative experiment (**A**) and pooled data from 3 experiments (**B**) are shown. Activated CIR- T cells were used as negative controls. (**C**) Median tumor sizes at Day 5, Day 10, and Day 15 post-infusion are indicated by horizontal bars along with individual tumor measurements represented by the data points. (**D**) We performed immunohistochemistry (top row 10x, bottom row 40x) to detect tumor infiltrating CD3+ T cells (arrows). (**E**) Routine H&E was used to assess the degree of tumor necrosis (asterisks) in mice treated with 1^st^ or 2^nd^ gen dTc, both with and without supplemental IL2 (left column 10x, right column 40x).

To determine the ability of anti-KIT dTc to traffic to, infiltrate, and limit growth of established tumor, we utilized a subcutaneous xenograft model. Human KIT+ GIST cells were injected subcutaneously into immunodeficient mice that were treated with tail vein injections of 1^st^ gen, 2^nd^ gen, or unmodified anti-KIT dTc 7 days later. Tumor measurements were performed in two dimensions (mm^2^) and are expressed as median percentage change relative to the tumor size on initial day of treatment. IL2 therapy was given along with dTc for some groups because IL2 is known to enhance T cell function and may play a role in tumor clearance. In a single repetition, significant reductions in tumor growth were mediated by 1^st^ gen dTc without IL2 (p=0.05) and 2^nd^ gen dTc (p<0.001) with IL2 (Figure 5A). When all data were pooled, both 1^st^ and 2^nd^ gen dTc had a significant impact on tumor growth in the absence of IL2 therapy. With IL2 support, 1^st^ gen dTc had a significant effect (p=0.05) while 2^nd^ gen dTc (p=0.13) demonstrated a favorable trend (Figure 5B). 1^st^ gen dTc may be more reliant on IL2 than 2^nd^ gen dTc because the presence of the co-stimulatory signal through the CD28 portion of the construct may reduce the dependence of 2^nd^ gen dTc on cytokines such as IL2. Data is further represented as tumor growth for each individual sample to demonstrate the range of values (Figure 5C). Following sacrifice, we harvested the tumors and confirmed the presence of adoptively transferred dTc and necrosis in animals treated with 1^st^ or 2^nd^ gen dTc (Figures 5D and E).

## Discussion

We have constructed a novel anti-KIT CIR that can be utilized to reprogram human T cells to recognize and kill KIT+ GIST tumors. The natural ligand for KIT, SCF, conferred anti-tumor specificity for the anti-KIT CIR and T cells were transduced with high levels of efficiency. Our *in vitro* studies confirmed that anti-KIT dTc proliferated and secreted IFNγ in response to two KIT+ GIST cell lines, one of which was established from a GIST clinically resistant to kinase-inhibitor therapy with imatinib. The dTc were also able to lyse KIT+ cells *in vitro*. Using a xenograft model, we demonstrated that systemic infusions of 1^st^ and 2^nd^ gen anti-KIT dTc resulted in significant reductions in tumor growth rates. Taken together, our initial study of anti-KIT dTc supports their further development as a novel treatment for KIT+ neoplasms, including those which have developed clinical resistance to kinase-inhibitor therapy.

Success with the clinical use of dTc has been reported for the treatment of chronic lymphocytic leukemia, melanoma, and synovial cell sarcoma [11,17]. DTc may offer a new therapeutic option for GIST treatment that can be used alone or in combination with other therapies, including TKIs. Imatinib has been reported to induce regulatory T cell apoptosis and its efficacy was enhanced by concurrent immunotherapy [18]. Adding imatinib to anti-KIT dTc infusions may augment efficacy through favorable immunomodulation within the tumor microenvironment, allowing the dTc to mediate enhanced tumor cell lysis.

Our histologic studies confirmed that anti-KIT dTc traffic to KIT+ tumors and mediate tumor necrosis after intravenous infusion. Few CTRL T cells were seen within the tumors following intravenous infusion, suggesting that either preferential trafficking or intratumoral proliferation accounted for the presence of anti-KIT dTc within the GIST xenografts. Although we demonstrated dTc within GIST xenografts, we speculate that the predominance of a central memory phenotype (CD62L+CCR7+CD45RO+) among anti-KIT dTc following production may have promoted preferential migration to lymphoid tissue, which we did not examine further [19,20]. Addition of IL-15 and IL-21 to promote an effector phenotype [21] may offer the potential of enhancing the efficacy of anti-KIT dTc. The optimal production method and dTc subsets for treatment of KIT+ tumors remain to be determined.

Significant delays in tumor growth following a single dTc infusion in our *in vivo* model support the potential utility of anti-KIT dTc for the treatment of GIST. Yet, only one animal experienced a complete regression and several factors may have limited *in vivo* activity of the anti-KIT dTc. Multiple infusions may be necessary to achieve complete regression of established tumors, given that the efficacy of the anti-KIT dTc seemed to wane 7 days following a single infusion. When comparing the median tumor sizes at several time points, a small number of outliers appeared to have a significant impact on the analysis of the 2^nd^ gen groups. In addition, the absence of a normal immune cell repertoire in the immunodeficient mice may have limited the extent of the anti-tumor effect following dTc infusion. We speculate that tumor cell lysis, antigen release, and the associated inflammatory response may stimulate endogenous immunity which may contribute to tumor regression. The implications of a normal endogenous immune system for the *in vivo* activity of anti-KIT dTc require clarification through additional studies.

Several limitations of our data warrant consideration. Our findings did not support a significant or consistent benefit to either the presence of CD28 signaling in the 2^nd^ gen dTc or IL-2 infusion for the treatment of established GIST xenografts. The addition of the CD28 moiety to other CIR constructs has been shown to enhance dTc persistence and lytic function, although conflicting results have been reported [7,22,23]. The absence of a compelling advantage for the 2^nd^ gen dTc compared to the 1^st^ gen in our model may be specific to the anti-KIT constructs and further testing will be required to explain the limited benefit of CD28 co-stimulation. *In vitro*, it has been shown that exogenous IL-2 was necessary in order for modified T cells to have a marked effect on tumor growth [23]. We speculate that immunodeficient mice did not show a significant response to supplemental IL-2 due to their lack of endogenous Treg that can deplete IL-2 from the environment [24]. Furthermore, Treg have been shown to directly inhibit the induction of IL-2 mRNA, which augments the requirement for exogenous IL-2 addition [17]. We are also unable to exclude the possibility that the modification of the T cells in some way altered their intrinsic responsiveness to IL-2 *in vivo*.

Finally, our *in vivo* murine model might not have permitted a full assessment of toxicity given that we utilized the human SCF moiety in the CIR. Expression of the KIT gene is essential for the development of normal hematopoiesis, proliferation, and migration of primordial germ cells and melanoblasts during embryogenesis as well as for the development of gastrointestinal pacemaker activity [16,25]. Although it may be reasonable to address this concern in a phase I trial with a small number of patients, development of suitable animal model prior to clinical studies would be preferable.

## Conclusions

We have successfully produced anti-KIT dTc and have demonstrated their capacity for recognizing and killing KIT+ GIST cells. The present study establishes the basis for further investigation of anti-KIT dTc for the treatment of advanced GIST refractory to tyrosine kinase inhibitors. Study of anti-KIT dTc function in the context of an intact immune system will be informative, along with more detailed characterization of the phenotype of the 1^st^ and 2^nd^ gen dTc. A phase I clinical trial will be required to demonstrate the safety of anti-KIT dTc in addition to their potential efficacy.

## Abbreviations

CIR: Chimeric immune receptor; CTRL: Untransduced T cells; CFSE: Carboxyfluorescein diacetate succinimidyl ester; dTc: Designer T cells; FBS: Fetal bovine serum; Gen: Generation; GIST: Gastrointestinal stromal tumor; IMDM: Iscove’s Modified Dulbecco’s Medium; KIT: CD117; KL: KIT-ligand; LDH: Lactate dehydrogenase; MFI: Mean fluorescence index; PBMC: Peripheral blood mononuclear cells; SCF: Stem cell factor; TIL: Tumor infiltrating lymphocytes; TKI: Tyrosine kinase inhibitor.

## Competing interests

The authors declare that they have no competing interests.

## Authors’ contributions

SCK - Conceived of the study and coordinated its design and execution, analyzed the data, wrote the manuscript, and created the figures. RB - Performed *in vivo* tumor experiments, and *in vitro* designer T cell production. Assisted with the writing of the manuscript, data analysis, and figure production. SN - Performed *in vitro* assays and contributed to the writing of the manuscript. LL - Performed *in vitro* assays and contributed to the writing of the manuscript. MT - Assisted with the writing and editing of the manuscript and figures. DO – Assisted with construct design, performed *in vitro* designer T cell production, and reviewed the figures and text. CN - Performed *in vitro* designer T cell production and assisted with *in vivo* experiments. NJE - Assisted with the writing and editing of the manuscript in addition to the study design. JAF - Assisted with writing of the manuscript, provided cell lines, and collaborated with the design and execution of *in vitro* and *in vivo* experiments. RPJ – Participated in study conception, chimeric antigen receptor design, data interpretation, and critically reviewed the figures and text. All authors read and approved the final manuscript.
